# Phosphate ions modulate enzyme activity and epistatic effects in two clavulanic acid‐resistant β‐lactamase mutants

**DOI:** 10.1002/pro.70325

**Published:** 2025-10-11

**Authors:** Marko Radojković, Saar F. Koene, Aleksandra Chikunova, Bogdan I. Florea, Sivanandam V. Natarajan, Aimee L. Boyle, Marcellus Ubbink

**Affiliations:** ^1^ Leiden Institute of Chemistry Leiden University Leiden The Netherlands; ^2^ School of Chemistry University of Bristol Bristol UK

**Keywords:** antibiotic resistance, beta‐lactamase, clavulanic acid, epistasis

## Abstract

Epistasis, the non‐additive effect of mutations, substantially undermines our ability to predict the evolutionary trajectories of enzymes. Epistatic effects are evident in the evolution of serine β‐lactamases, where synergistic mutations can enhance antimicrobial resistance. We recently demonstrated that positive epistasis drives clavulanic acid resistance in double‐mutant libraries of the β‐lactamase BlaC. Here, we employed various biochemical and structural approaches to investigate molecular mechanisms underlying epistasis in the fitness of two double mutant variants, I105Y‐S130G and I105G‐G132N. For the latter enzyme, epistatic compensation of catalytic activity was detected for multiple substrates and proved to be highly buffer‐dependent. Non‐additive effects were also evident in the thermostability profile of the I105G‐G132N variant. The interplay between the reduced clavulanic acid sensitivity of the S130G and G132N variants and active‐site modifications induced by Ile105 substitutions is discussed. The results demonstrate that the origins of epistasis can be rooted in multiple enzyme traits, highlighting its important role in the evolution of antimicrobial resistance.

## 1 INTRODUCTION

Enzymes evolve in response to changes in the environment of their host. This process of evolutionary adaptation occurs via mutations that have a neutral or positive impact on their fitness. Since the structure–function relationship of enzymes depends on delicate amino acid interactions, non‐additive mutational effects can often lead to unexpected phenotypes, a phenomenon known as epistasis (Miton et al., 2021; Miton & Tokuriki, 2016; Østman et al., 2012; Phillips, 2008; Starr & Thornton, 2016). Positive epistasis, in which the impact of combined mutations is greater than the sum of individual contributions, can enable access to new mutational pathways (Breen et al., 2012; Lunzer et al., 2010; Schenk et al., 2013; Weinreich et al., 2006). These non‐additive effects can stem from adjusting the precise positioning of active site residues, thereby fine‐tuning substrate binding and enhancing catalysis (Blazeck et al., 2022; Heckmann et al., 2018; Johnston et al., 2024; Noor et al., 2012). Epistasis can also arise from mutations that alter conformational dynamics, which can substantially improve enzymatic activity (Campbell et al., 2018; Gonzalez et al., 2016; Ortlund et al., 2007; Otten et al., 2020; Rossi et al., 2022).

Non‐additive interactions are often key drivers of the evolution of β‐lactamases and, consequently, antibiotic resistance development (Dabos et al., 2025; Mira et al., 2021; Weinreich et al., 2006; Widmann & Pleiss, 2014). In a recent article on an OXA‐48 (Ambler class D (Ambler et al., 1991)) variant with four mutations, a change in the rate‐limiting step of ceftazidime hydrolysis resulted in a 40‐fold increase in bacterial resistance, despite marginal contributions of individual mutations (<2 fold) (Fröhlich et al., 2024). Deep mutational scanning of pairwise mutations across 17 active site positions of CTX‐M‐14 β‐lactamase (Ambler class A) exposed several hotspots that mediate epistasis through enhanced substrate interactions (Judge et al., 2024). These studies provided invaluable insights into the mechanistic determinants of epistasis concerning the activity on cephalosporin and penicillin β‐lactams. However, little is known about the role of combinatorial mutations in the creation of inhibitor‐resistant bacterial phenotypes. It is important to determine whether these mutations can give rise to epistatic effects and to identify which biochemical and biophysical properties are amenable to non‐additive changes, as inhibitor‐resistant β‐lactamases frequently occur in clinical isolates of patients (Knox, 1995; Prinarakis et al., 1997; Tamma & Munita, 2024).

Here, we investigate how epistatic interactions influence the phenotypes of the two double mutant variants I105Y‐S130G and I105G‐G132N of *Mycobacterium tuberculosis* class A β‐lactamase, BlaC, which previously showed greatly improved fitness against the combination of carbenicillin and inhibitor, clavulanic acid, in a cellular assay (Radojković & Ubbink, 2024). Fitness in this case means the compromise between the ability of the enzyme to evade inhibition and retention of a sufficient level of activity to hydrolyze the antibiotic. The residue at position 105, known as “gatekeeper” (Feiler et al., 2013), is in most class A β‐lactamases an amino acid with an aromatic side chain, which yields high activity against penicillin‐like β‐lactams (Doucet et al., 2004; Judge et al., 2023; Papp‐Wallace et al., 2010). The residues at positions 130 and 132 have been associated with reduced sensitivity to clavulanic acid for BlaC variants (Egesborg et al., 2015; Kurz et al., 2013; Soroka et al., 2015). Despite these two positions being very close in sequence, they exhibit different effects on clavulanic acid sensitivity upon substitution. Ser130 is a conserved residue among class A β‐lactamases and plays an important role in substrate binding and proton transfer during β‐lactam ring opening (Atanasov et al., 2000; Helfand et al., 2003). Moreover, it is involved in irreversible crosslinking of clavulanate and other mechanism‐based inhibitors (Thomas et al., 2005). The mutation S130G leads to the loss of activity for β‐lactam antibiotics and to slower inactivation of the enzyme by clavulanic acid (Egesborg et al., 2015). In contrast, the substitution G132N prevents inactivation by clavulanate by increasing the rate of hydrolysis (Soroka et al., 2015). Interestingly, this was only observed for BlaC, as other class A β‐lactamases already have Asn at this position but do not exhibit the same behavior (Ishii et al., 2007; Luhavaya & Grigorenko, 2010; Payne et al., 1994).

In this work, kinetic profiling of all double and single mutant enzymes revealed notable epistatic compensation for carbenicillin hydrolysis conferred by the I105G substitution. The I105Y‐S130G variant showed high bacterial resistance against the combination of clavulanic acid and carbenicillin. Based on crystallographic data, we provide a rationale for the functional relevance of the I105Y and S130G mutations. Furthermore, we show that the presence of phosphate ions can dramatically alter enzyme activity and that mechanisms of clavulanate resistance can depend on the presence of anions in the buffer. Our findings help to unravel aspects of the complex nature of epistasis, potentially enhancing our ability to predict the evolution of β‐lactamases.

## 2 RESULTS

### 2.1 Mutations of the gatekeeper residue lead to a positive epistatic compensation of activity loss

**FIGURE 1:**
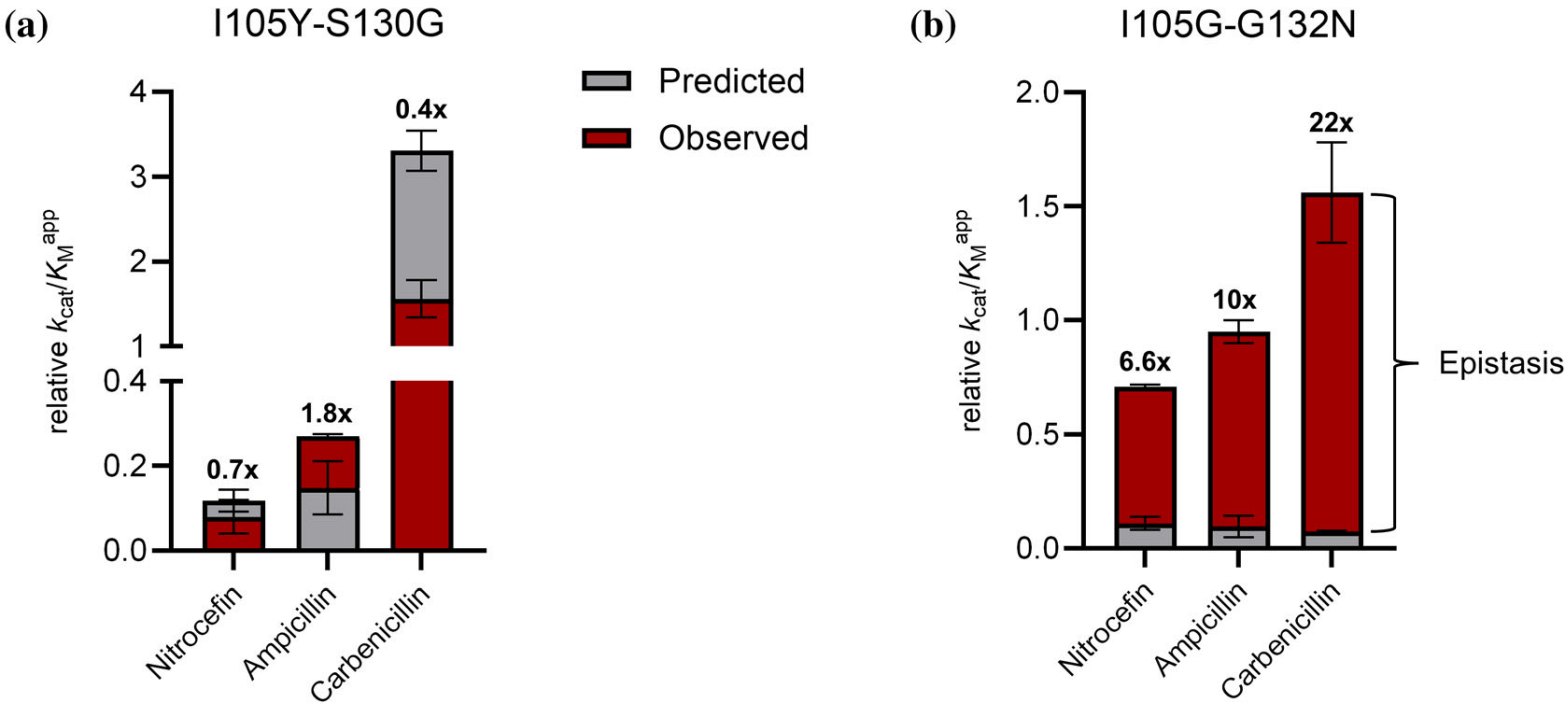
Epistasis in enzyme activity. Relative catalytic efficiency (*k*_cat_/*K*_M_^app^) of the double mutants is plotted against predicted relative catalytic efficiencies from the *k*_cat_/*K*_M_^app^ values of the single mutants. If the difference is nonzero (including errors), epistasis occurs (see Methods for detailed description). Numbers above bars represent fold differences between observed and predicted catalytic efficiencies, with values >1 indicating positive epistasis, and values <1 indicating negative epistasis. (a) BlaC I105Y‐S130G; (b) BlaC I105G‐G132N.

Alteration in enzyme activity has been proposed as a key determinant in the evolutionary adaptation of β‐lactamases (Fröhlich et al., 2024; Knies et al., 2017; Singh & Dominy, 2012). To test whether changes in catalytic efficiency are also responsible for the fitness compensation, we determined steady‐state kinetic parameters of purified double mutant variants for three substrates and compared them to the activity profiles of their corresponding single mutants (Table 1). Both S130G and G132N substitutions lead to reduced catalytic efficiency for all three tested substrates (nitrocefin, ampicillin, and carbenicillin). Generally, mutation S130G has a more detrimental impact on activity than G132N and leads to an approximately 30‐fold decrease in *k*_cat_/*K*_M_^app^ values for nitrocefin and ampicillin, compared to the wild‐type. Under the experimental conditions used (phosphate buffer), the addition of mutation I105Y to BlaC S130G enhances activity, and this compensation is reflected in the elevated turnover numbers of the double mutant. The compensatory effect was even more pronounced in the case of the I105G‐G132N variant, for which I105G entirely neutralizes the activity loss of G132N (up to 19‐fold increase in catalytic efficiency). To determine whether this activity compensation is epistatic, we compared the relative *k*_cat_/*K*_M_^app^ values of the double mutants to the product of the relative *k*_cat_/*K*_M_^app^ values of the single mutants according to Equation (1) (Østman et al., 2012; Radojković & Ubbink, 2024):

(1) $$\varepsilon_{\mathrm{AB}}={\mathrm{CE}}_{\mathrm{AB}}-{\mathrm{CE}}_{A}\times {\mathrm{CE}}_{B}>0$$

where *ε*_AB_ stands for positive pairwise epistasis, and CE_AB_, CE_A_, and CE_B_ for relative catalytic efficiency of the double mutant and corresponding single mutants, respectively. If the product of CE_A_ and CE_B_ is lower than the observed CE_AB_, positive epistasis occurs (see Materials & Methods for a detailed description). As shown in Figure 1, BlaC I105Y‐S130G only exhibits positive epistasis for ampicillin, whereas for BlaC I105G‐G132N, epistasis is detected for all three tested substrates. Furthermore, the magnitude of epistasis observed for the latter variant is substantially higher than that of BlaC I105Y‐S130G, suggesting a stronger synergy between these two residues. The individual *k*_cat_ and *K*_M_^app^ values of I105G‐G132N and its single mutants (Table 1) suggest that epistasis is primarily governed by the non‐additive effects in *K*_M_^app^ values for ampicillin and carbenicillin, rather than changes in the turnover number.

**TABLE 1:** Steady‐state kinetic parameters determined for wild‐type (WT) BlaC and various mutants.

	*K*_M_^app^ (μM)	*k*_cat_ (s^−1^)	*k*_cat_/*K*_M_^app^ (μM^−1^ s^−1^)	*k*_cat_ */K*_M_^app^ relative to the wild‐type
Nitrocefin				
WT	180 ± 70	90 ± 20	0.5 ± 0.2^a^	1
I105Y	18 ± 1	39 ± 10	2.1 ± 0.1	4.2 ± 0.2
S130G	358 ± 4	5 ± 1	0.014 ± 0.003	0.03 ± 0.01
I105Y‐S130G	500 ± 200	21 ± 7	0.04 ± 0.02	0.08 ± 0.04
I105G	240 ± 40	79 ± 5	0.34 ± 0.02	0.68 ± 0.04
G132N	210 ± 30	18 ± 2	0.08 ± 0.02	0.16 ± 0.04
I105G‐G132N	115 ± 1	41 ± 1	0.357 ± 0.005	0.71 ± 0.01
Ampicillin				
WT (Radojković, Chikunova, et al., 2025)	68 ± 3	14 ± 2	0.21 ± 0.01^a^	1
I105Y	49 ± 1	48 ± 1	0.98 ± 0.02	4.43 ± 0.1
S130G	240	1.7 ± 1	0.007 ± 0.001	0.03 ± 0.01
I105Y‐S130G	296 ± 6	17 ± 1	0.057 ± 0.001	0.270 ± 0.005
I105G	19 ± 1	3.95 ± 0.04	0.21 ± 0.01	1.00 ± 0.05
G132N	1800 ± 800	33 ± 12	0.02 ± 0.01	0.10 ± 0.05
I105G‐G132N	93 ± 1	18 ± 1	0.20 ± 0.01	0.95 ± 0.05
Carbenicillin				
WT (Radojković, Chikunova, et al., 2025)	5.5 ± 0.7	0.51 ± 0.02	0.09 ± 0.01^a^	1
I105Y	1.5 ± 1	2.0 ± 0.1	1.34 ± 0.07	14.9 ± 0.8
S130G	72 ± 5	1.2 ± 0.1	0.020 ± 0.001	0.22 ± 0.01
I105Y‐S130G	21 ± 3	2.7 ± 0.2	0.13 ± 0.02	1.4 ± 0.2
I105G	1.5 ± 0.4	0.12 ± 0.01	0.081 ± 0.004	0.90 ± 0.04
G132N	224 ± 11	1.62 ± 0.02	0.0072 ± 0.0004	0.080 ± 0.004
I105G‐G132N	7.0 ± 0.9	0.96 ± 0.07	0.14 ± 0.02	1.6 ± 0.2

*Note*: All measurements were done in phosphate buffer (100 mM NaPi, pH 6.4) at 25°C. Errors represent one standard deviation of the mean of triplicate measurements. The *K*_M_ is indicated as apparent because regular Michaelis–Menten kinetics do not apply to the two‐step hydrolysis reaction (Sun et al., 2023). Michaelis–Menten plots for all substrates are shown in Figure S1.

^a^ The WT error of *k*_cat_/*K*_M_^app^ values was not used for the error propagation of the relative *k*_cat_/*K*_M_^app^ values of the other variants.

### 2.2 Epistasis is present in the thermostability profile of I105G‐G132N

**FIGURE 2:**
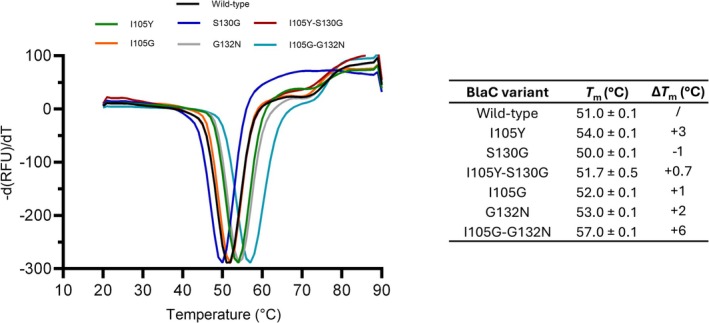
Melting curves of wild‐type BlaC and all double and single mutant variants. (Left) The negative first derivative of the normalized fluorescence signal; (right) melting temperatures for each variant. Errors represent one standard deviation of the mean of the triplicate measurements.

Mutations of β‐lactamases that result in function gain often come at the expense of thermostability (Baig et al., 2014; Mehta et al., 2015; Thomas et al., 2010), and the same was observed for BlaC variants with reduced clavulanic acid sensitivity (Egesborg et al., 2015). We wanted to test whether this is true for the mutants examined in this study by assessing the thermostability profiles of all double and single‐mutant enzymes. Hence, melting temperatures (*T*_m_) were determined from the thermal unfolding in the presence of SYPRO orange dye. Surprisingly, most of the substitutions positively impact stability (Figure 2). The *T*_m_ value of the I105Y‐S130G variant was 1.3°C lower than the sum of the effects of the individual mutations (+3 and −1°C for I105Y and S130G, respectively), indicative of negative epistasis. Conversely, the combination of I105G and G132N mutations leads to a remarkable 6°C increase in stability, which is twice as much as expected from the individual contributions (+1 and +2°C for I105G and G132N, respectively). This unusual observation suggests that positive epistasis can also originate from the non‐additive improvements in enzyme stability.

### 2.3 In vitro inhibitor susceptibility indicates negative epistasis

**FIGURE 3:**
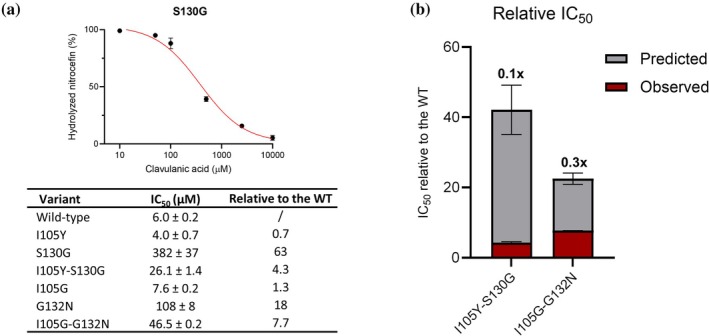
In vitro clavulanic acid susceptibility. (a) (Top panel) The amount of hydrolyzed nitrocefin after 10 min in the presence of different clavulanic acid concentrations is expressed relative to the control (no inhibitor). These values were plotted against increasing inhibitor concentration, and sigmoidal fitting was performed to obtain the IC_50_ value. Shown as an example are the results for BlaC S130G (sigmoidal graphs of all variants can be found in Figure S2). (Bottom panel) IC_50_ values of all BlaC variants. All measurements were done in 100 mM NaPi buffer, pH 6.4, at 25°C. The enzyme concentration was 2 nM, and the nitrocefin concentration was 125 μM. (b) Relative IC_50_ values of the double mutants plotted against relative IC_50_ values predicted from the product of the single mutants. If the difference is nonzero (including errors), epistasis occurs (see Methods for detailed description). Numbers above bars represent fold differences between observed and predicted IC_50_, with values <1 indicating negative epistasis. The error bars represent one standard error of the duplicate measurement.

Next, we investigated the potential of all single and double mutant variants to evade clavulanic acid inhibition in vitro and compared them to the inhibition profile of the wild‐type enzyme (Figure 3). To that end, nitrocefin hydrolysis in the presence of various clavulanate concentrations was monitored for a fixed period of 10 min. Subsequently, the total amount of hydrolyzed nitrocefin was plotted against clavulanic acid concentrations, and IC_50_ values were derived from sigmoidal fittings (Figures 3a and S2). The S130G variant showed the lowest susceptibility for the inhibitor, resulting in a 63 times increase in IC_50_ value, compared to the wild‐type, followed by a G132N (18‐fold increase). Both double mutants also showed reduced clavulanate sensitivity, although to a much lower extent (4.3‐fold and 7.7‐fold increase, for I105Y‐S130G and I105G‐G132N, respectively) than predicted, indicating negative epistasis (Equation 1, Figure 3b).

### 2.4 The S130G mutation impairs recovery of the double mutant from clavulanic acid inhibition

**FIGURE 4:**
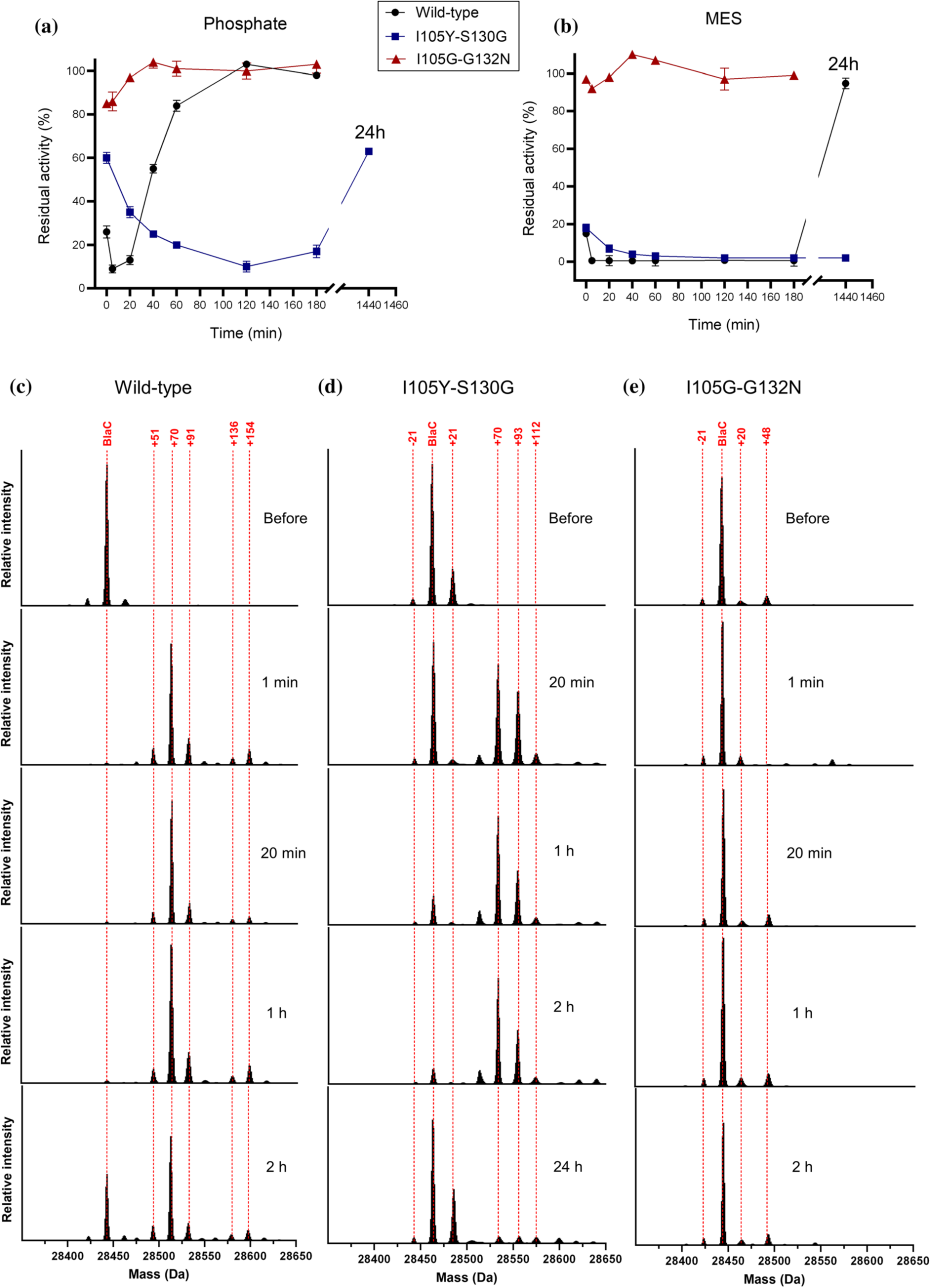
Time‐dependent inactivation and recovery from clavulanic acid inhibition. (a, b) Residual activity of BlaC variants for nitrocefin (100 μM) after incubation with clavulanic acid for different time periods. (a) In phosphate buffer (100 mM NaPi, pH 6.4); (b) in MES buffer (100 mM MES, pH 6.4). Errors represent one standard deviation of triplicate measurements. (c–e) Charge‐deconvoluted mass spectra of BlaC variants after incubation with clavulanic acid in phosphate buffer. Upon inhibition, covalently bound adducts appear in the spectra. After prolonged incubation, the enzyme returns to its free form. (c) WT; (d) I105Y‐S130G. (e) I105G‐G132N, the enzyme stays in the free form during the whole incubation time, indicating turnover of the inhibitor. Enzyme‐to‐inhibitor ratio was the same in both experiments (20 μM:200 μM). For the activity measurements, aliquots were taken and diluted to an enzyme concentration of 20 nM, and the substrate was added.

To further explore the resistance mechanism of the I105Y‐S130G and I105G‐G132N enzymes, time‐dependent inactivation and recovery assays were performed. Herein, the clavulanate concentration was kept constant, and the residual activities of the enzymes were measured at different time points. Recovery of wild‐type BlaC from clavulanic acid inhibition is known to be greatly accelerated in the presence of phosphate ions (Elings et al., 2017). We wondered whether double mutants would be affected in the same manner and conducted experiments in both phosphate and MES buffers (Figure 4a,b). Wild‐type BlaC gets fully inhibited quickly (<5 min), irrespective of the buffer environment. After 2 h of incubation, the wild‐type enzyme reaches its original activity again in the phosphate buffer (Figure 4a). BlaC I105Y‐S130G gets inhibited slowly and is fully inhibited after 2 h in phosphate buffer. After 24 h of incubation, it only recovers to 63% of the control activity in the phosphate buffer and stays fully inhibited in the MES buffer. The I105G‐G132N enzyme showed initially a slight drop in activity, followed by a swift recovery in both buffers.

Additionally, we monitored the inhibition intermediates and recovery of the resting state enzyme in phosphate buffer using whole‐protein mass spectrometry (MS, Figure 4c–e). In the MS spectrum of wild‐type BlaC (Figure 4c), a +70 Da peak becomes the dominant species already after 1 min of incubation. This species is believed to correspond to the propionaldehyde ester intermediate (Figure S3i). Multiple other peaks emerge, including +51, +91, +136, and +154. The first peak was mentioned previously (Elings et al., 2017), possibly being an artifact of the sample treatment and MS analysis, although we do not exclude the possibility of cross‐linking with Ser130, which is expected to yield a peak with a mass of +52. The remaining three peaks likely correspond to hydrated propionaldehyde (+88, Figure S3k), and the decarboxylated imine (+155, Figure S3e) and its dehydrated form (+137, Figure S3h) (Hugonnet & Blanchard, 2007; Tremblay et al., 2008). After 2 h, the wild‐type enzyme has regained full activity in the recovery assay, but the +70 peak remains present in the MS analysis. This discrepancy could be a consequence of the experimental setup. To perform the activity assay, the reaction mixture needs to be diluted 1000‐fold, effectively removing the clavulanic acid, and this takes several minutes. For an enzyme with rapid clavulanate turnover in the phosphate buffer, this can lead to hydrolysis of the remaining clavulanate adduct, yielding an active enzyme. For the MS analysis, the enzyme did not have this opportunity to recover in the absence of clavulanic acid. The MS analysis of BlaC I105Y‐S130G and I105G‐G132N mirrored the observations of the recovery assays, showing slow inhibition and recovery for the former and no inhibition for the latter (Figure 4d,e). In the case of BlaC I105G‐G132N, complete hydrolysis of the acyl‐enzyme intermediate likely happens very fast (<1 min), as previously observed for G132N (Soroka et al., 2015).

### 2.5 Bacterial resistance to clavulanic acid is enhanced by double mutants

Subsequently, we determined MIC values for clavulanic acid and carbenicillin of *E. coli* cells producing all single and double mutant variants to probe if the detected epistatic effects observed in the in vitro characterization correlate with bacterial resistance levels (Table 2). BlaC I105Y showed the highest resistance to carbenicillin (Table 2), in line with the high in vitro activity (Table 1). The MIC value of the G132N variant for carbenicillin was only two times lower than that of the wild‐type, whereas the catalytic efficiency showed a 13 times decrease (Table 1). Consequently, the I105G‐G132N double mutant did not display a MIC that reflected the large positive epistasis for carbenicillin activity observed in vitro (Figure 1).

**TABLE 2:** MIC values for carbenicillin and clavulanic acid of WT and other BlaC variants.

Variant	MIC carbenicillin (μg/mL)	MIC clavulanic acid^a^ (μg/mL)
pUK21^b^	10	—
Wild‐type	800	1
I105Y	6400	2
S130G	100	4
I105Y‐S130G	800	32
I105G	800	4
G132N	400	1
I105G‐G132N	800	16

^a^ In combination with carbenicillin at a concentration of 50 μg/mL.

^b^ pUK21 vector without *blaC* gene.

The I105Y‐S130G variant showed the strongest resistance to clavulanic acid, leading to a highly resistant bacterial phenotype with a 32‐fold increase in the MIC in comparison to the wild‐type. Interestingly, the MIC values of both double mutants are 4‐fold higher than expected from the individual mutations, demonstrating positive epistasis—contrary to the negative epistasis suggested by in vitro inhibition.

### 2.6 Epistasis shows buffer dependency

The discrepancy between in vitro activity and MIC value of the G132N variant for carbenicillin raised the question about the possible buffer effect on substrate conversion, so we evaluated the in vitro activity of all variants in the MES buffer. Unfortunately, the carbenicillin conversion in the MES buffer could not be followed due to the high substrate background, so ampicillin was used instead. Indeed, the activities differ substantially, with BlaC S130G exhibiting roughly 5‐fold and G132N 10‐fold higher activity in MES than in phosphate buffer (Table S1). Thereupon, the positive epistasis observed for the ampicillin activity of I105Y‐S130G enzyme in phosphate buffer even flipped to negative in MES buffer (Figure S4a), whereas the magnitude of positive epistasis detected for I105G‐G132N diminished by a factor of 7.4 in MES buffer (Figure S4b).

### 2.7 Structural implications of the mutated residues

**FIGURE 5:**
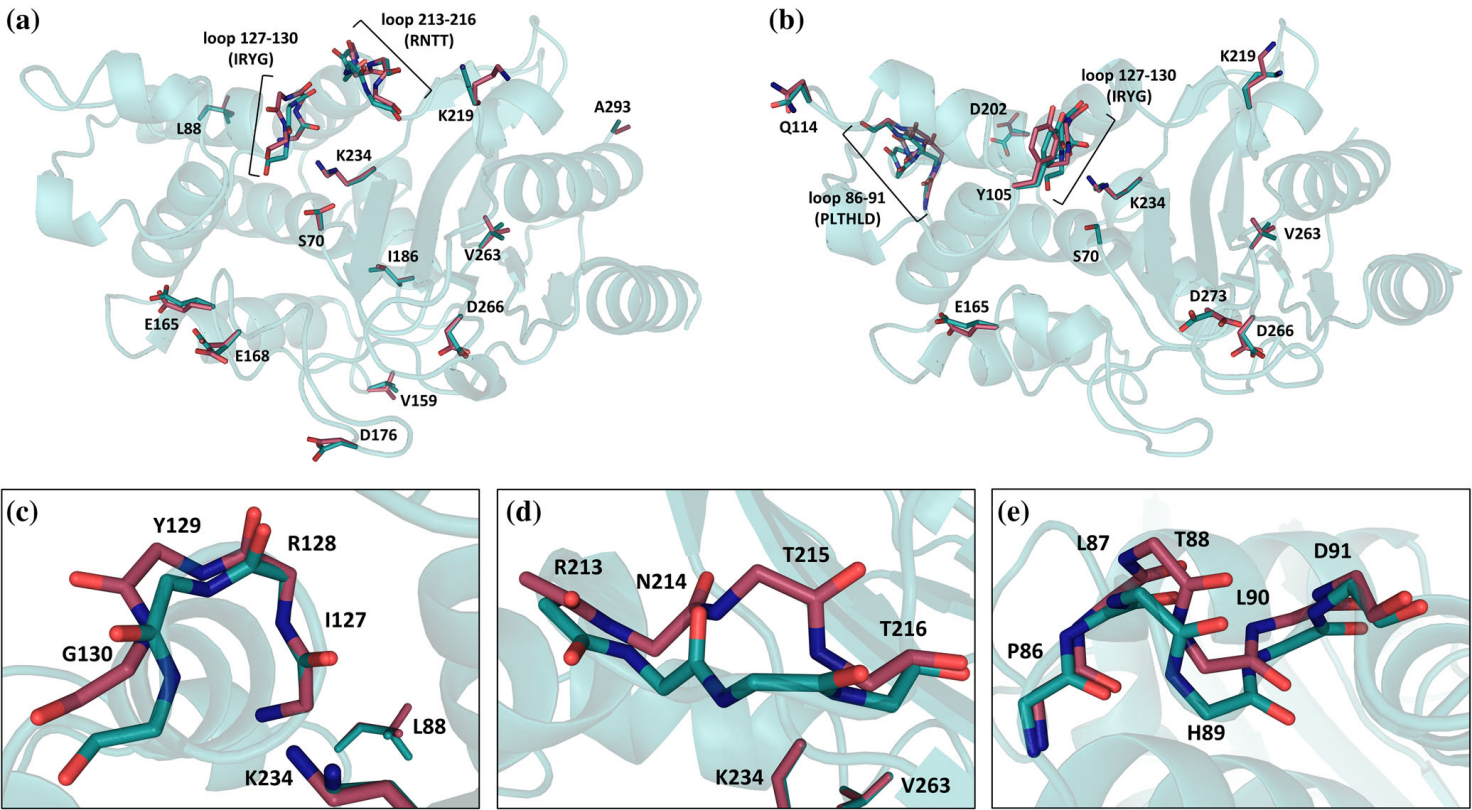
Crystal structure of BlaC I105Y‐S130G (PDB 9QI3) with double conformations for multiple residues. (a) Chain A; (b) chain B. Note that the double conformation of loop 127–130 is modeled in both chains. (c–e) Detail showing the loops modeled in double conformations. (c) Loop 127–130 (chain A); (d) loop 213–216 (chain A); and (e) loop 86–91 (chain B).

To understand the structure–function relationship of the studied variants, the crystallization of both purified double mutant enzymes was attempted. Despite several rounds of buffer optimization and seeding, protein crystals with good diffraction properties could not be acquired for the I105G‐G132N variant. The protein crystals of the I105Y‐S130G enzyme diffracted to 1.3‐Å resolution with *R*_work_/*R*_free_ factor values of 14% and 17% (Table S2). Two enzyme molecules were modeled in a unit cell with multiple residues adopting two conformations (Figure 5a,b). The double conformation of the 127–130 backbone loop, harboring the mutation site, could be modeled in both chains, whereas loops containing the residues 213–216 and 86–91 could be modeled in two orientations only in one of the chains (Figure 5c–e, respectively). The side chains of numerous other residues were also modeled in double conformations, including Ser70 and Tyr105.

**FIGURE 6:**
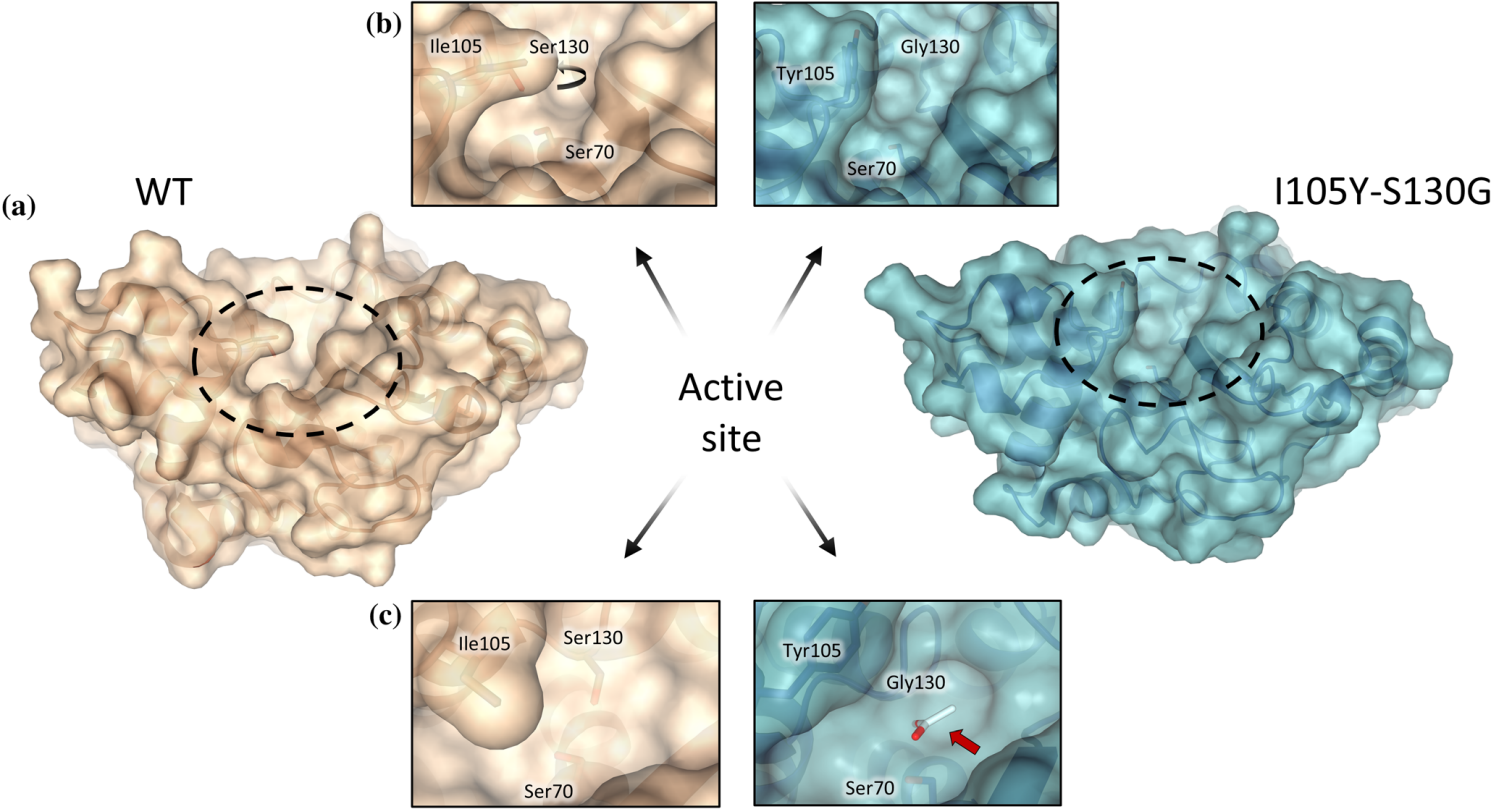
Active site comparison between BlaC wild‐type (PDB 2GDN (Wang et al., 2006)) and I105Y‐S130G. (a) Surface representation of the structures. (b) Local environment of residue 105 showing broadened access to the active site bestowed by the Ile to Tyr substitution. The round black arrow indicates the position of residue 130, which is not visible from this angle. (c) Active site from the top perspective. The red arrow shows the acetate ion present at the position of the missing Ser130 side chain. The acetate molecule is shown as a white stick, with oxygen atoms colored red.

The comparison between the crystal structures of BlaC wild‐type and I105Y‐S130G revealed two differences in the active site (Figure 6). First, the substitution of Ile to Tyr at position 105 considerably widens access to the active site, due to the orientation of the Tyr side chain (Figure 6b), which resembles the conformation found in the TEM‐1 enzyme (Strynadka et al., 1992). Second, in the place of the missing Ser130 side chain in BlaC I105Y‐S130G, an acetate ion is present (Figure 6c), suggesting possible anion‐assisted substrate hydrolysis. We probed this hypothesis by monitoring nitrocefin hydrolysis in the buffer with different acetate and phosphate concentrations. As shown in Table S3, some increase in enzyme activity is observed with increased acetate concentrations. However, a large concentration of another anion, phosphate, seems to leave the *k*_cat_ unaffected and to raise the *K*_M_. Thus, the suggestion remains speculative.

To see if the dynamic behavior inferred from the crystal structure would be detectable in the solution, we used nuclear magnetic resonance (NMR) spectroscopy. To that end, two‐dimensional (2D) transverse relaxation optimized heteronuclear single quantum coherence (TROSY‐HSQC) spectra were recorded for ^15^N labeled BlaC I105Y‐S130G and I105G‐G132N (Figure S5). Most of the backbone H‐N moieties could be assigned from the previously obtained data for wild‐type BlaC (Elings et al., 2017; van Alen et al., 2023). Additional assignments were done using three‐dimensional HNCa spectra on ^15^N‐^13^C labeled samples, resulting in a confident assignment of 245/251 non‐proline backbone amides for I105Y‐S130G, and 249/251 for I105G‐G132N. Interestingly, the amide of residue 130, which could not be assigned in the wild‐type spectrum, was present in the spectrum of both double mutants. The spectrum of BlaC I105Y‐S130G did not provide evidence for dynamic behavior. Some possible peak splitting was observed, but it remains unclear whether they belong to unassigned amides or represent two states of certain amides (Figure S5a). Likewise, the spectrum of I105G‐G132N did not indicate any dynamic behavior in the slow regime (<100 s^−1^, Figure S5b). Additionally, the millisecond dynamics of both enzymes were investigated with Carr‐Purcell‐Meiboom‐Gill (CPMG) relaxation dispersion experiments. However, no significant dispersion effects were observed.

**FIGURE 7:**
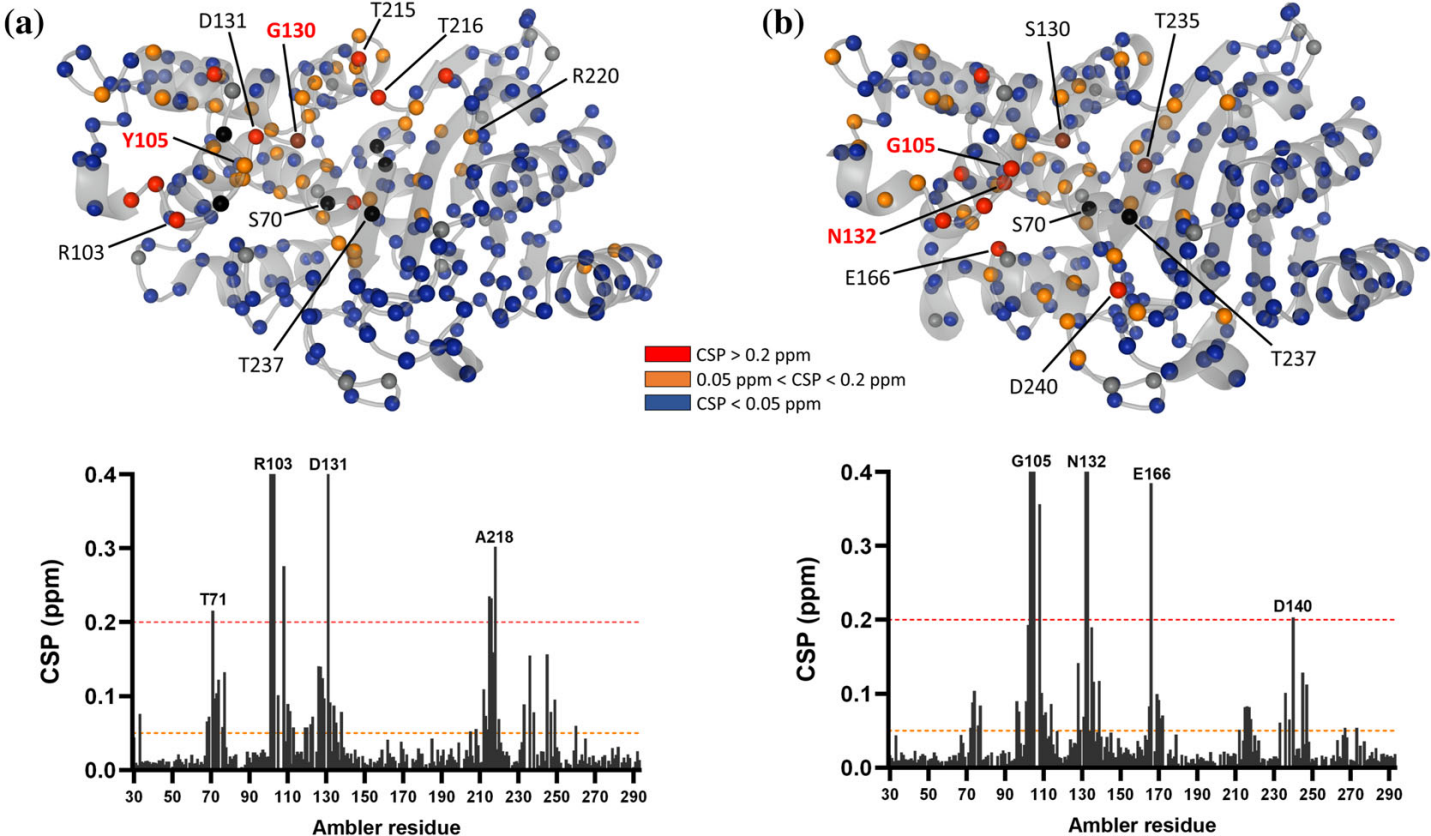
Chemical shift perturbations (CSP) of backbone amide resonances upon mutations. (a) BlaC I105Y‐S130G and (b) BlaC I105G‐G132N. The CSPs are plotted with the color code on the 3D structure (upper part; PDB 9QI3 and 2GDN (Wang et al., 2006) for I105Y‐S130G and I105G‐G132N, respectively) and as bar graphs per Ambler residue (lower part). Red, CSP of ≥0.2 ppm; orange, 0.2 ppm > CSP ≥ 0.05 ppm; blue, CSP of <0.05 ppm; black, residues could not be assigned or broadened beyond detection; brown, residues present in the mutant, but not wild‐type spectrum; gray, proline residues. Dashed lines on the bar graphs represent cut‐offs for different CSP groups as defined by the color scheme.

The backbone amide chemical shifts of both double mutants were compared to those of the wild‐type BlaC (Figure 7). Both spectra show modest chemical shift perturbations (CSPs) for most amides, suggesting only minor structural changes. Nevertheless, the CSPs are widespread (Figure 7, top). For BlaC I105Y‐S130G, the biggest CSPs were present in the active site and around mutated residues (Figure 7a, bottom). The residues comprising the 213–216 loop also experience large CSPs, which may correlate with the double conformation observed in the crystal structure (Figure 5d). The CSPs of BlaC I105G‐G132N are slightly more dispersed over the structure (Figure 7b, top). Interestingly, besides large CSPs detected around the mutation sites, Glu166, a residue critical for the deacylation step of the substrate hydrolysis, also displays a large shift.

## 3 DISCUSSION

Despite the tremendous success of directed evolution over the past decades, inferring the relationship between an enzyme's fitness and its amino acid sequence remains difficult. The major reason for this problem lies in epistasis, which describes non‐additive effects when mutations are combined, resulting in an unpredictable phenotype (Phillips, 2008; Starr & Thornton, 2016). Therefore, deciphering this non‐additivity has become one of the major challenges in current enzyme engineering efforts. While many studies captured patterns of epistasis by characterizing enzyme libraries with deep sequencing (Buda et al., 2023; Gonzalez & Ostermeier, 2019; Johnston et al., 2024; Parera & Martinez, 2014; Radojković & Ubbink, 2024; Steinberg & Ostermeier, 2016), there is a lack of literature explaining which enzyme features drive its occurrence. The prevailing opinion is, however, that non‐additive improvements in enzyme activity are a primary source of epistasis (Fröhlich et al., 2024; Heckmann et al., 2018; Parera & Martinez, 2014; Zhang et al., 2012).

In this study, we investigated the factors underlying epistasis in the phenotypes of two inhibitor‐resistant BlaC double mutants, I105Y‐S130G and I105G‐G132N. These variants had previously demonstrated notably enhanced fitness in the presence of both carbenicillin and clavulanic acid (Radojković & Ubbink, 2024). In this context, fitness refers to the ability of the enzyme to resist inhibition while maintaining sufficient activity to hydrolyze the antibiotic. Here, we aimed to disentangle the fitness contributions of the individual mutations by using detailed in vitro characterization.

The S130G mutation abolishes inactivation by clavulanate (Figure 3), but also substantially lessens catalytic efficiency toward carbenicillin (Table 1). We note that this effect is buffer dependent because the *k*_cat_ for ampicillin is 8‐fold lower than for wild‐type BlaC in phosphate buffer, but 2.5 times higher in MES buffer (Table S1). The introduction of the I105Y substitution restores activity for all substrates (in phosphate buffer) by increasing the turnover number, with the compensation being primarily non‐epistatic (Figure 1). The increase in *k*_cat_ presumably comes from favorable edge‐to‐face stacking interactions of the Tyr105 side chain with the benzyl or thienyl moiety of the substrate, locking it in an optimal position during catalysis (Chen & Herzberg, 2001; Díaz et al., 2003; Doucet et al., 2004). Since clavulanic acid does not have an aromatic group, this stabilizing effect is likely less pronounced. However, the increase in the size of the active site cavity rendered by the I105Y mutation (Figure 6a) seems to increase inhibitor susceptibility (Figure 3), contrary to what we observed for BlaC I105R (Radojković, Chikunova, et al., 2025). Although the IC_50_ value of the I105Y‐S130G mutant is notably lower than expected (negative epistasis, Figure 3b), the MIC for clavulanic acid suggests a synergistic effect (positive epistasis) of the two mutations in the cellular context (Table 2). This apparent discrepancy likely stems from the different substrates in the two assays (nitrocefin vs. carbenicillin) and the inherent complexity of biological systems, where factors such as antibiotic and inhibitor uptake rates and interactions with cellular components significantly influence the MIC phenotype. The high buffer dependency mentioned above indicates how strongly the activity depends on the environmental conditions.

**FIGURE 8:**
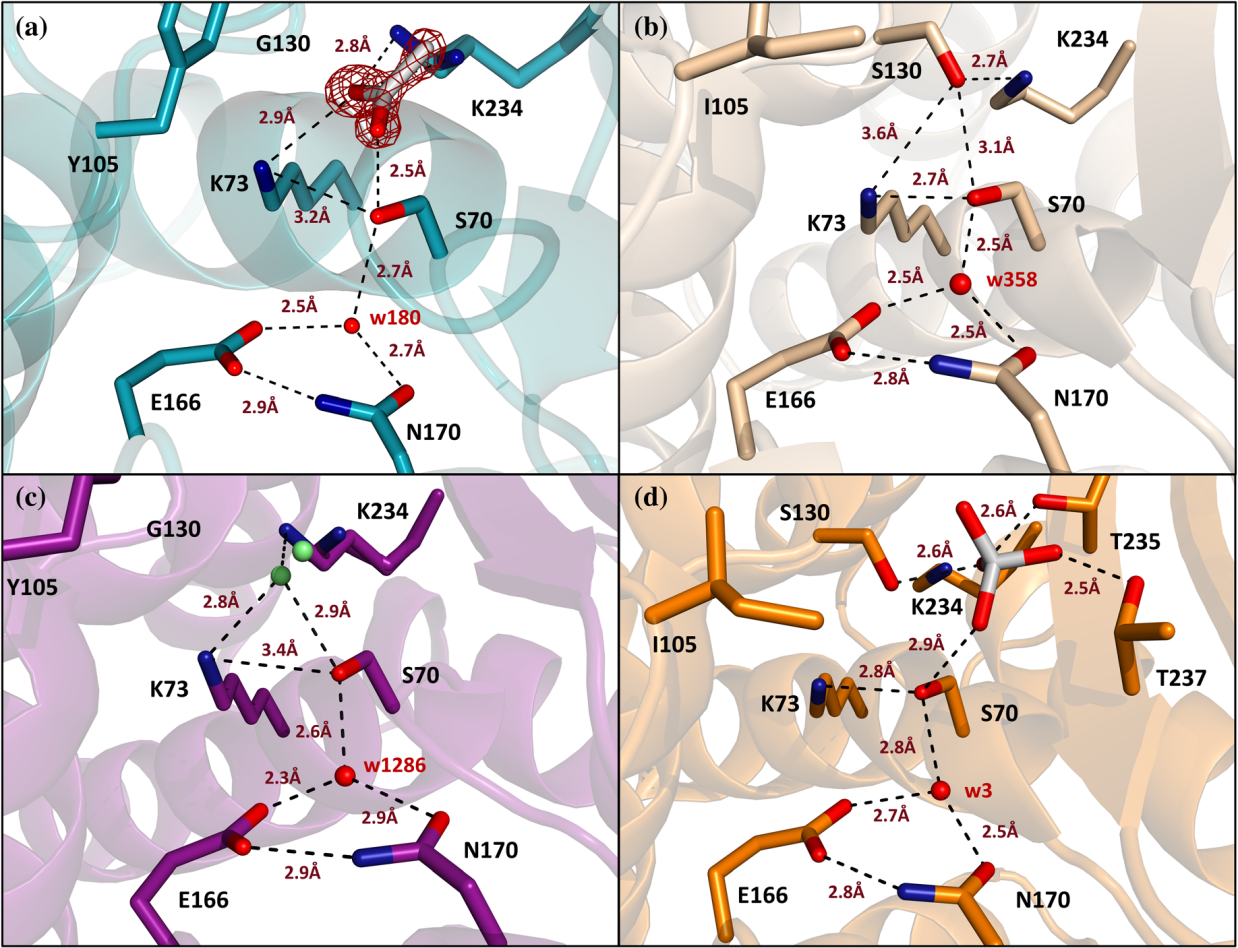
The active site configuration of different class A β‐lactamases. (a) BlaC I105Y‐S130G (cyan, 9QI3); (b) BlaC wild‐type (wheat, 2GDN (Wang et al., 2006)); (c) TEM‐76, which possesses the S130G mutation (magenta, 1YT4 (Thomas et al., 2005)); (d) BlaC wild‐type with bound phosphate (orange, 5NJ2 (Elings et al., 2017)). The conserved catalytic water is present in all four instances and shown in red. (a) In the absence of the Ser130 side chain in the I105Y‐S130G structure, an acetate ion occupies its space. The 2*mF*_o_ − *DF*_c_ electron density map (1.85*σ*) is centered around the acetate ion, which is shown as sticks. (c) In TEM‐76, two new water molecules (green) were observed, one of which occupies the same position as the hydroxyl group of Ser130 and has been proposed to replace its role in substrate hydrolysis. (d) A phosphate ion occupies the carboxylate binding pocket in the BlaC wild‐type structure. Side chains of the active site residues and anions are shown as sticks. Possible H‐bonds are indicated with dashed lines, with the distances given in Å.

The presence of an acetate ion in the crystal structure of I105Y‐S130 implied a possible anion‐assisted hydrolysis (Figure 6c), so we looked closely at the positioning of the key catalytic residues and water molecules in the active site (Figure 8a). The overall configuration looks similar to that of the wild‐type (Figure 8b), with the acetate molecule occupying the position of the missing Ser130 side chain and engaging in H‐bonding with Ser70, Lys73, and Lys234. Curiously, in the crystal structure of TEM‐76 (Thomas et al., 2005), the same space is occupied by two additional water molecules (Figure 8c). The authors propose that the new water molecules serve as an alternative to Ser130 for protonation of the β‐lactam nitrogen and ring opening during substrate hydrolysis. Our kinetic data indicate that acetate ions could potentially also take over this role, as the 4‐fold increase in acetate concentration augmented I105Y‐S130G activity by 64% (Table S3). Interestingly, raising the concentration of phosphate anions led to the opposite effect and a 3‐fold decrease in enzyme activity (Table S3). The presence of phosphate ions also strongly affected the time‐dependent inactivation and recovery from the clavulanic acid inhibition (Figure 4), similar to what was demonstrated before for the wild‐type enzyme (Elings et al., 2017). In comparison to the wild‐type, BlaC I105Y‐S130G took more time to be fully inhibited but also recovered more slowly (Figure 4a,b). This points to an important role of Ser130 in the recovery mechanism, which likely aids in the correct positioning of the phosphate molecule (Figure 8d), as noted previously (Elings et al., 2017). It was proposed that the phosphate could either perform the nucleophilic attack on the carbon of the covalently bound adduct on its own or donate a proton to Ser130, which simultaneously protonates Ser70 after the nucleophilic water attack in the deacylation step. In either scenario, the S130G mutation would disrupt the clavulanate recovery promoted by the phosphate ions.

The conformational flexibility indicated by the crystal structure obtained at cryogenic conditions could not be confirmed by the NMR data recorded at room temperature (25°C), at least not on the examined s^−1^ and ms^−1^ scales. The enzyme either exhibits dynamic behavior on a faster timescale (ns^−1^) or the observed double conformations are present only in the crystals, for example, induced by the moderately low pH in the crystallization buffer (pH 4.5, Table S2).

The determined MIC value for the carbenicillin of BlaC G132N did not correlate with the in vitro steady‐state parameters (Tables 1 and 2, respectively), as the displayed bacterial phenotype is better (MIC 2 times lower than the wild‐type) than assumed from the relative catalytic efficiency of the isolated enzymes (*k*_cat_/*K*_M_^app^ value 13 times lower than the wild‐type). As with BlaC S130G, the kinetic parameters were highly buffer dependent, showing 10 times higher catalytic efficiency for ampicillin in MES than in phosphate buffer (Table S1). Although both *k*_cat_ and *K*_M_^app^ are ameliorated in MES buffer, the effect on *K*_M_^app^ is larger, suggesting high affinity of the G132N enzyme for phosphate, which likely competes with substrate molecules for binding of the carboxy group in the carboxy binding pocket, as suggested previously (Elings et al., 2017). A similar increase in the affinity has been reported for the open form of BlaC P167S (Sun et al., 2023).

In phosphate buffer, the mutation I105G fully compensated for the activity loss caused by G132N across the three tested substrates (Table 1), yielding a 22‐fold enhancement of catalytic efficiency relative to the expected additive effects (Table 1, Figure 1b). This epistatic compensation primarily comes from non‐additive improvements in *K*_M_^app^ values, especially in the case of penicillin‐like substrates, likely by improving access to the active site in the absence of the side chain of residue 105. Nevertheless, additional structural investigation is necessary to explain the observed effects. The bacterial resistance profile of the I105G‐G132N variant for clavulanic acid was much better than expected from the individual variants (Table 2). A possible explanation could be higher cellular protein levels, as the double mutant showed a remarkable +6°C increase in *T*_m_ (Figure 2). Together with preserved activity for carbenicillin and enhanced clavulanate hydrolysis, this leads to a strong MIC phenotype (Table 2).

To summarize, we have shown that the magnitude of epistasis can be highly dependent on the buffer environment. It is worth further exploring how the regulation of other environmental factors, such as temperature, pH, and ionic strength, influences epistasis. Furthermore, we underline the important role of residue 105 in the creation of highly resistant bacterial variants, as the same position was found to be a major epistatic compensator for cefotaxime in double‐mutant libraries of CTX‐M‐14 β‐lactamase (Judge et al., 2024). Disentangling the epistatically enhanced fitness of inhibitor‐resistant variants may be necessary to understand how such phenotypes arise and ultimately improve drug development strategies.

## 4 MATERIALS AND METHODS

### 4.1 Site‐directed mutagenesis

Mutations were introduced into the *blaC* gene by the QuikChange method (Agilent) and verified by Sanger sequencing. All mutagenic primers are listed in Table S4.

### 4.2 Protein production and purification

Recombinant proteins were produced in *E. coli* BL21 (DE3) pLysS cells transformed with pET28a plasmids carrying the *blaC* gene with an N‐terminal His tag and TEV cleavage site (Figure S6) (Radojković, van Ingen, et al., 2025). Protein production and purification were performed as described previously (Elings et al., 2017), except for the size exclusion chromatography step, which was omitted. The purity of protein samples was assessed with SDS‐PAGE (≥95%). Pure protein samples were aliquoted, flash‐frozen in liquid nitrogen, and stored at –80°C in 100 mM sodium phosphate buffer (pH 6.4) or 100 mM MES buffer (pH 6.4).

### 4.3 Enzyme kinetics

Steady‐state kinetic parameters were determined using nitrocefin (BioVision), ampicillin (Serva), and carbenicillin (Sigma‐Aldrich) as substrates. Measurements were performed in triplicate in a 10 mm QS High Precision cell (Hellma Analytics), using a PerkinElmer Lambda 1050+ UV–Vis spectrometer thermostated at 25°C. Nitrocefin conversion was monitored at 486 nm (Δ*ε* = 11,300 M^−1^ cm^−1^) for 3 min in the presence of 2 nM BlaC. Ampicillin hydrolysis was followed at 235 nm (Δ*ε* = 861 M^−1^ cm^−1^) for 3 min in the presence of 10 nM BlaC. Carbenicillin degradation was recorded at 235 nm (Δε = 938 M^‐1^ cm^‐1^) for 3 min with 50 nM BlaC. The substrate concentration was varied from 10 to 300 μM. Measurements were performed in 25–200 mM phosphate buffer (pH 6.4), 100 mM MES buffer (pH 6.4), and 25–100 mM sodium acetate buffer (pH 4.8). GraphPad Prism 10.1 was used to fit initial reaction velocities to the Michaelis–Menten equation (Equation 2):

(2) $$v_{i}=\frac{k_{\mathrm{cat}}\left[E\right]\left[S\right]}{K_{M}^{\mathrm{app}}+\left[S\right]}$$

where *v*_*i*_ is the initial reaction velocity, [*S*] is the initial substrate concentration, [E] is the total enzyme concentration, and *k*_cat_ and *K*_M_^app^ are the Michaelis–Menten parameters. The *K*_M_ is indicated as apparent because regular Michaelis–Menten kinetics do not apply to the two‐step hydrolysis reaction (Sun et al., 2023). In the case of two‐phased kinetic behavior, slopes of the first phase were fitted to Equation (2). Michaelis–Menten plots of all analyzed variants for all three substrates are shown in Figure S1.

### 4.4 Examination of epistatic effects in enzyme activity

The activity compensation was determined to be epistatic based on Equation (3):

(3) $$\varepsilon_{\mathrm{AB}}={\mathrm{CE}}_{\mathrm{AB}}-\left({\mathrm{CE}}_{A}\times {\mathrm{CE}}_{B}+∆{\mathrm{CE}}_{\mathrm{AB}}+∆{\mathrm{CE}}_{A\times B}\right)>0$$

where *ε*_AB_ is a positive pairwise epistasis, CE_AB_, CE_A_, and CE_B_ are relative catalytic efficiencies of the double mutant and corresponding single mutants, ΔCE_AB_ is the error in the relative catalytic efficiency of the double mutant, and ΔCE_A×B_ is the propagated error of the relative catalytic efficiencies of the single mutants (see Equation 4). If the observed relative activity of the double mutant (CE_AB_) is higher than the sum of the predicted relative activity (CE_A_ × *CE*_B_) and their corresponding errors (ΔCE_AB_ + ΔCE_A×B_), positive epistasis occurs.

(4) $$\Delta {\mathrm{CE}}_{A\times B}={\mathrm{CE}}_{A}\times {\mathrm{CE}}_{B}\sqrt{\;{\left(\frac{{\mathrm{\Delta CE}}_{A}}{{\mathrm{CE}}_{A}}\right)}^{2}+{\left(\frac{{\mathrm{\Delta CE}}_{B}}{{\mathrm{CE}}_{B}}\right)}^{2}}$$

### 4.5 Thermal shift assay

The thermal stability of the proteins was assessed with a thermal shift assay using a CFX96 Touch Real‐time PCR Detection System (Bio‐Rad). The fluorescence signal of SYPRO Orange dye was followed in the presence of 10 μM BlaC in 100 mM phosphate buffer (pH 6.4). The temperature was increased from 20 to 90°C with a 1°C increment, and the samples were incubated for 1 min at each temperature before detection of the fluorescence signal. Melting temperatures (*T*_m_) were determined from the derivatives of the obtained curves. Errors in the reported values represent one standard deviation of the mean of triplicate measurements.

### 4.6 Determination of IC_50_ for clavulanic acid

Inhibition assays of all purified enzymes (2 nM) were performed in the presence of various clavulanic acid concentrations (0.25 μM to 10 mM) and a fixed nitrocefin concentration (125 μM) in phosphate buffer (100 mM NaPi, pH 6.4) at 25°C. The absorbance at 486 nm was followed for 10 min, and the amount of the hydrolyzed product was expressed relative to the control (no inhibitor). These values were then plotted against increasing inhibitor concentration, and GraphPad Prism 10.1 was used to fit a sigmoidal function and derive IC_50_ values (Figure S2). To examine epistasis, the relative IC_50_ (compared to the wild‐type) values of double mutants were compared to the product of the relative IC_50_ values of the single mutants (see Equation 3; CE = IC_50_). Errors represent one standard error of the duplicate measurements.

### 4.7 Inhibition recovery

Time‐dependent inactivation and recovery from clavulanic acid inhibition were carried out by incubating 20 μM enzyme with 200 μM clavulanic acid in phosphate buffer (100 mM NaPi, pH 6.4) or MES buffer (100 mM MES, pH 6.4) at 25°C. At each time point, activity measurements were performed by dilution in buffer without the inhibitor to a final concentration of 20 nM BlaC with 100 μM nitrocefin. The reaction was monitored for 3 min, and the residual activity was determined by comparing the final concentration of the formed product to the control experiment (BlaC without clavulanic acid). Errors represent one standard deviation of triplicate measurements.

### 4.8 Mass spectrometry

Protein samples were flash‐frozen in liquid nitrogen at different time points during incubation with clavulanic acid in 100 mM NaPi, pH 6.4, at 25°C (20 μM:200 μM, enzyme‐to‐inhibitor ratio). After thawing, the sample buffer was exchanged for 10 mM ammonium acetate, pH 6.8, using Micro Bio‐Spin chromatography columns (Bio‐Rad). Samples were then loaded onto a Silica‐C4 reversed phase UPLC column and analyzed using a Synapt G2‐Si mass spectrometer (Waters), 15–30 min after thawing. Data were deconvoluted for charge using MaxEnt 1 software (Waters).

### 4.9 Minimum inhibitory concentration determinations

The MIC was determined for *E. coli* KA797 cells carrying various pUK21‐*blaC* plasmids with the *lac* promoter and *tat*‐signal peptide upstream of the *blaC* gene (Figure S7) (Radojković, van Ingen, et al., 2025). Overnight cultures of *E. coli* were inoculated into fresh LB medium with 50 μg/mL kanamycin and incubated at 37°C until OD_600_ reached 2. Subsequently, cells were pipetted into a honeycomb 100‐well plate with the selection antibiotic (carbenicillin or carbenicillin with clavulanic acid) to OD_600_ = 0.01. The concentration of the selection antibiotic was increased in 2‐fold increments. The plate was incubated for 16 h at 37°C in a Bioscreen C Pro plate reader with continuous shaking, and the OD_600_ was recorded every 15 min. The MIC was defined as the concentration of the selection antibiotic at which no increase in OD_600_ was observed.

### 4.10 Crystallization

Crystallization conditions for BlaC I105Y‐S130G (14 mg/mL) and I105G‐G132N (11 mg/mL) were screened for by the sitting‐drop method using the BCS and Morpheus (Molecular Dimensions, Catcliffe, UK) screens at 20°C with 200 nL drops with a 1:1 protein to screening condition ratio. Based on initial hits in the form of needles, the crystal growth was further optimized by varying the concentrations of sodium acetate, zinc chloride, and pH, yielding suitable crystals for BlaC I105Y‐S130G within a month. Crystals were mounted on cryo‐loops in mother liquor with an additional 25% glycerol for cryoprotection. The exact formulation of the conditions can be found in Table S2.

### 4.11 X‐ray data collection and structure solving

Diffraction data for BlaC I105Y‐S130G crystals were collected at the European Synchrotron Radiation Facility (Grenoble, France) with a wavelength of 0.87 Å on a Dectris Eiger2_9M detector. The resolution cutoff was determined based on completeness and CC1/2 values. The data were scaled using Aimless (Evans, 2011). The structure was solved by molecular replacement using MOLREP from the CCP4 suite (Winn et al., 2011) and by using PDB entry 2GDN (Wang et al., 2006) as a search model. Two protein molecules were modeled in an asymmetric unit. Subsequently, building and refinement were performed using Coot and REFMAC (Winn et al., 2011). The final refinement was performed with the PDB REDO web server (Joosten et al., 2014; Sobolev et al., 2020). Structure validation showed a RamaZ (Sobolev et al., 2020) score of 0.081; 98% of all residues are within the Ramachandran plot favored regions, with residues Cys69 and Arg220 being the outliers. Data collection and refinement statistics can be found in Table S2.

### 4.12 Nuclear magnetic resonance spectroscopy experiments

TROSY‐HSQC and HNCA spectra were recorded at 25°C using a Bruker AVIII HD 850 MHz spectrometer equipped with a TCI cryoprobe. The samples contained ∼0.6 mM [^15^N] or ∼0.4 mM [^13^C,^15^N] BlaC I105Y‐S130G, and samples contained ∼0.7 mM [^15^N] or ∼0.5 mM [^13^C,^15^N] BlaC I105G‐G132N in 100 mM sodium phosphate (pH 6.4) and 8% D2O. Data were processed with Topspin 4.0.7 (Bruker Biospin) and analyzed using CCPNmr Analysis V2.4.2 (Vranken et al., 2005). Spectra were compared to the HSQC and HNCA spectra of the wild‐type BlaC (Elings et al., 2017; van Alen et al., 2023), and average chemical shift perturbations (CSPs, Δ*δ*) of backbone amides were calculated using Equation 5:

(5) $$\Delta \delta =\sqrt{\frac{1}{2}\;\left[\Delta \delta_{1}+{\left(\frac{{\Delta \delta }_{2}}{5}\right)}^{2}\right]}$$

where Δ*δ*_1_ and Δ*δ*_2_ are the differences in chemical shifts in ppm in spectra of the mutant and wild‐type enzymes for ^1^H and ^15^N, respectively. Overlays of ^1^H‐^15^N TROSY‐HSQC spectra for both double mutants and the wild‐type enzyme are shown in Figure S5.

## AUTHOR CONTRIBUTIONS

**Marko Radojković:** Conceptualization; investigation; methodology; writing – review and editing; writing – original draft; formal analysis; project administration; visualization. **Saar F. Koene:** Investigation; visualization; formal analysis; conceptualization; project administration; methodology. **Aleksandra Chikunova:** Investigation; formal analysis. **Bogdan I. Florea:** Investigation. **Sivanandam V. Natarajan:** Investigation. **Aimee L. Boyle:** Writing – review and editing; supervision. **Marcellus Ubbink:** Conceptualization; funding acquisition; writing – review and editing; methodology; project administration; supervision; resources.

## Supporting information


**APPENDIX S1:** Supplementary information.

## Data Availability

The data that supports the findings of this study are available in the supplementary material of this article.
