# Supplementary material for: Phosphate ions modulate enzyme activity and epistatic effects in two clavulanic acid‐resistant β‐lactamase mutants
Source: Protein Sci. 2025 Oct 11;34(11):e70325. doi: 10.1002/pro.70325 (PMC12514841; doi:10.1002/pro.70325)
Supplement: Supplementary file 1 — APPENDIX S1: Supplementary information. [file PRO-34-e70325-s001.pdf]

Supplementary Information for

Phosphate ions modulate enzyme activity and epistatic effects in two clavulanic acid-resistant β-lactamase mutants

Marko Radojković[1], Saar F. Koene[1], Aleksandra Chikunova[1], Bogdan I. Florea[1], Sivanandam V. Natarajan[1], Aimee L. Boyle[1,2] and Marcellus Ubbink[1,*]

[1]Leiden Institute of Chemistry, Leiden University, Einsteinweg 55, 2333 CC Leiden, The Netherlands
[2]School of Chemistry, University of Bristol, BS8 1TS, Bristol, United Kingdom

*Corresponding author, e-mail: m.ubbink@chem.leidenuniv.nl

Supplementary Tables

Supplementary Figures

Supplementary References

SUPPLEMENTARY TABLES

Table S1. Apparent Michaelis-Menten kinetic parameters for ampicillin hydrolysis. Reactions were carried out in 100 mM phosphate (pH 6.4) and 100 mM MES buffer (pH 6.4) at 25 °C. Errors represent one standard deviation of the mean of triplicate measurements. Michaelis-Menten plots of all variants in both buffers are shown in Figure S1.

	Ampicillin phosphate			
	K_M^{app} (μM)	k_{cat} (s^{-1})	k_{cat}/K_M^{app} ($\mu M^{-1} s^{-1}$)	k_{cat}/K_M^{app} relative to the wild-type
WT [1]	68 ± 3	14 ± 2	0.21 ± 0.01[†]	1
I105Y	49 ± 1	48 ± 1	0.98 ± 0.02	4.43 ± 0.1
S130G	240	1.7 ± 1	0.007 ± 0.001	0.03 ± 0.01
I105Y-S130G	296 ± 6	17 ± 1	0.057 ± 0.001	0.270 ± 0.005
I105G	19 ± 1	3.95 ± 0.04	0.21 ± 0.01	1.00 ± 0.05
G132N	1800 ± 800	33 ± 12	0.02 ± 0.01	0.10 ± 0.05
I105G-G132N	93 ± 1	18 ± 1	0.20 ± 0.01	0.95 ± 0.05
	**Ampicillin MES**			
	K_M^{app} (μM)	k_{cat} (s^{-1})	k_{cat}/K_M^{app} ($\mu M^{-1} s^{-1}$)	k_{cat}/K_M^{app} relative to the wild-type
WT	46 ± 12	9 ± 1	0.21 ± 0.01	1
I105Y	89 ± 7	28 ± 1	0.32 ± 0.03	1.52 ± 0.14
S130G	588	21 ± 1	0.035 ± 0.001	0.165 ± 0.005
I105Y-S130G	813 ± 132	23 ± 3	0.029 ± 0.006	0.14 ± 0.03
I105G	31 ± 6	6.0 ± 0.4	0.21 ± 0.04	1.00 ± 0.19
G132N	282 ± 9	61 ± 1	0.22 ± 0.01	1.04 ± 0.05
I105G-G132N	183 ± 21	54 ± 2	0.30 ± 0.04	1.42 ± 0.05

Table S2. Crystallization conditions, data collection, and refinement statistics for BlaC I105Y-S130G structure.

**PDB ID**	9QI3
Conditions	0.015 M ZnCl2<br>22 %v/v PEGSM<br>0.13 M Na Acet, pH 4.5
Resolution (Å)	135.18-1.30 (1.32-1.30)
Space group	P212121
Unit cell *a*, *b*, *c* (Å)	39.44, 41.42, 269.62
α, β, γ	90, 90, 90
CC1/2	97.2 (79.0)
R pim (%)	5.8 (29)
<*I*/σ>	12.2 (2.3)
Completeness (%)	99.7 (99.9)
Multiplicity	1.9 (1.9)
Unique reflections	109981 (5438)
**Refinement**	
Atoms: protein/ions/ligands/water	4328/1/26/675
B-factors: protein/ions/ligands/water ($Å^2$)	13/22/27/26
Rwork/Rfree (%)	14/17
Bond lengths RMSZ/RMSD (Å)	0.926/0.0244
Bond angles RMSZ/RMSD (°)	1.104/1.92
Ramachandran plot preferred/outliers	489/4
RamaZ score	0.08
Clash score	8.44
MolProbity score (percentile)	1.46 (79th)

Table S3. Apparent Michaelis-Menten kinetic parameters of I105Y-S130G enzyme for nitrocefin hydrolysis. Reactions were performed in 25-100 mM sodium acetate (pH 4.8) and 25-200 mM sodium phosphate (NaPi) buffer (pH 6.4) at 25 °C. Nitrocefin hydrolysis in acetate buffer displayed two phases, of which the second was substrate concentration-independent in the measured range. The provided kinetic parameters are derived from the slopes of the initial phase. The apparent K_M^{app} is high, and only k_{cat}/K_M^{app} could be provided. N.d. – not determined. Michaelis-Menten plots for all conditions are shown in Figure S1.

	Nitrocefin		
	K_M^{app} (μM)	k_{cat} (s^{-1})	k_{cat}/K_M^{app} ($\mu M^{-1} s^{-1}$)
I105Y-S130G (25 mM acetate)	n.d.	n.d.	$(5.3 \pm 0.7) \times 10^{-4}$
I105Y-S130G (50 mM acetate)	n.d.	n.d.	$(5.9 \pm 0.9) \times 10^{-4}$
I105Y-S130G (100 mM acetate)	n.d.	n.d.	$(8.7 \pm 0.4) \times 10^{-4}$
I105Y-S130G (25 mM NaPi)	104 ± 5	13 ± 1	$(12.9 \pm 1.4) \times 10^{-2}$
I105Y-S130G (100 mM NaPi)	500 ± 200	21 ± 7	$(4 \pm 2) \times 10^{-2}$
I105Y-S130G (200 mM NaPi)	362 ± 137	13 ± 4	$(3.8 \pm 1.8) \times 10^{-2}$

Table S4. Primer sequences used in this study.

**Site-directed mutagenesis primers**	
I105Y_F	GTGATGATATCCGTAGCTATAGTCCGGTTGCACAG
I105Y_R	CTGTGCAACCGGACTATAGCTACGGATATCATCAC
S130G_F	GCAGCAATTCGTTATGGTGATGGCACCGCAGCC
S130G_R	GGCTGCGGTGCCATCACCATAACGAATTGCTGC
I105G_F	GTGATGATATCCGTAGCGGTAGTCCGGTTGCACAG
I105G_R	CTGTGCAACCGGACTACCGCTACGGATATCATCAC
G132N_F	CAATTCGTTATAGTGATAACACCGCAGCCAATCTG
G132N_R	CAGATTGGCTGCGGTGTTATCACTATAACGAATTG

SUPPLEMENTARY FIGURES

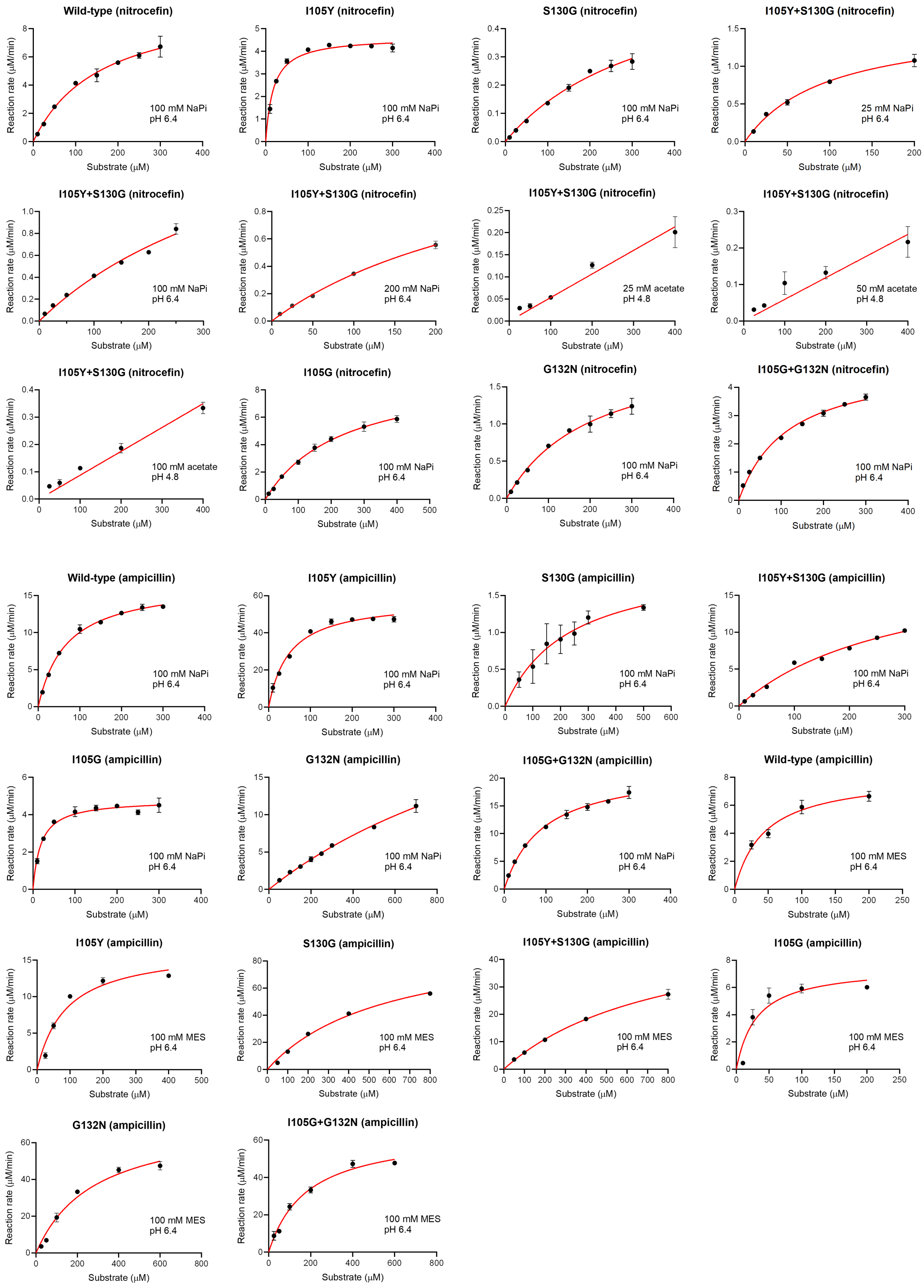

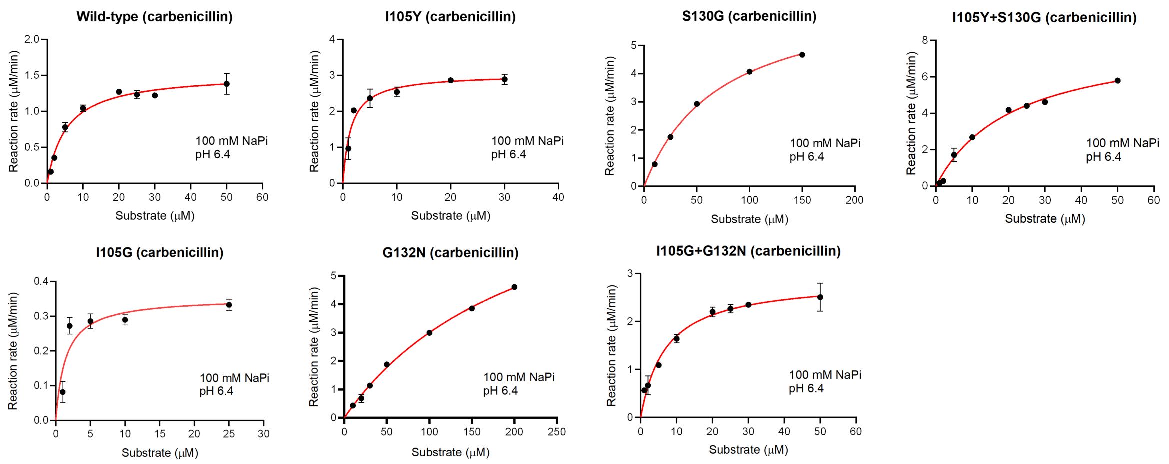

Figure S1. Michaelis-Menten plots of all BlaC variants characterized in this study using nitrocefin, ampicillin, and carbenicillin as substrates. The red line represents a non-linear or linear fit to the experimental data points. Errors represent one standard deviation of the triplicate measurements.

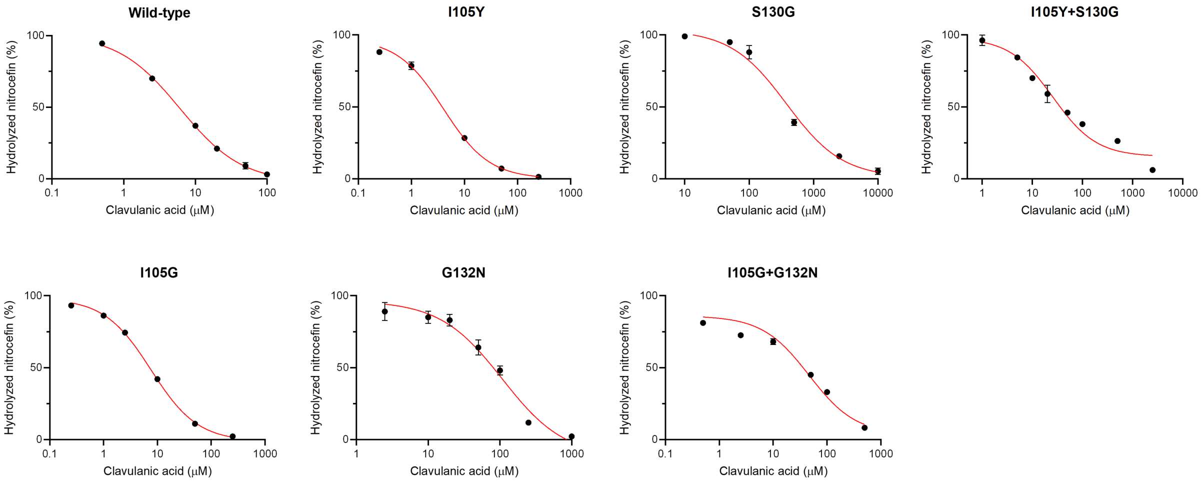

Figure S2. IC_{50} determined from clavulanic acid susceptibility assays. The amount of hydrolyzed nitrocefin after 10 min in the presence of different clavulanic acid concentrations is expressed relative to the control (no inhibitor). These values were plotted against increasing inhibitor concentration, and sigmoidal fittings were performed to obtain the IC_{50} values. Note that the x-axis values are given on a log scale. All measurements were done in 100 mM NaPi buffer, pH 6.4, at 25 °C. The enzyme concentration was 2 nM, and the nitrocefin concentration was 125 μM.

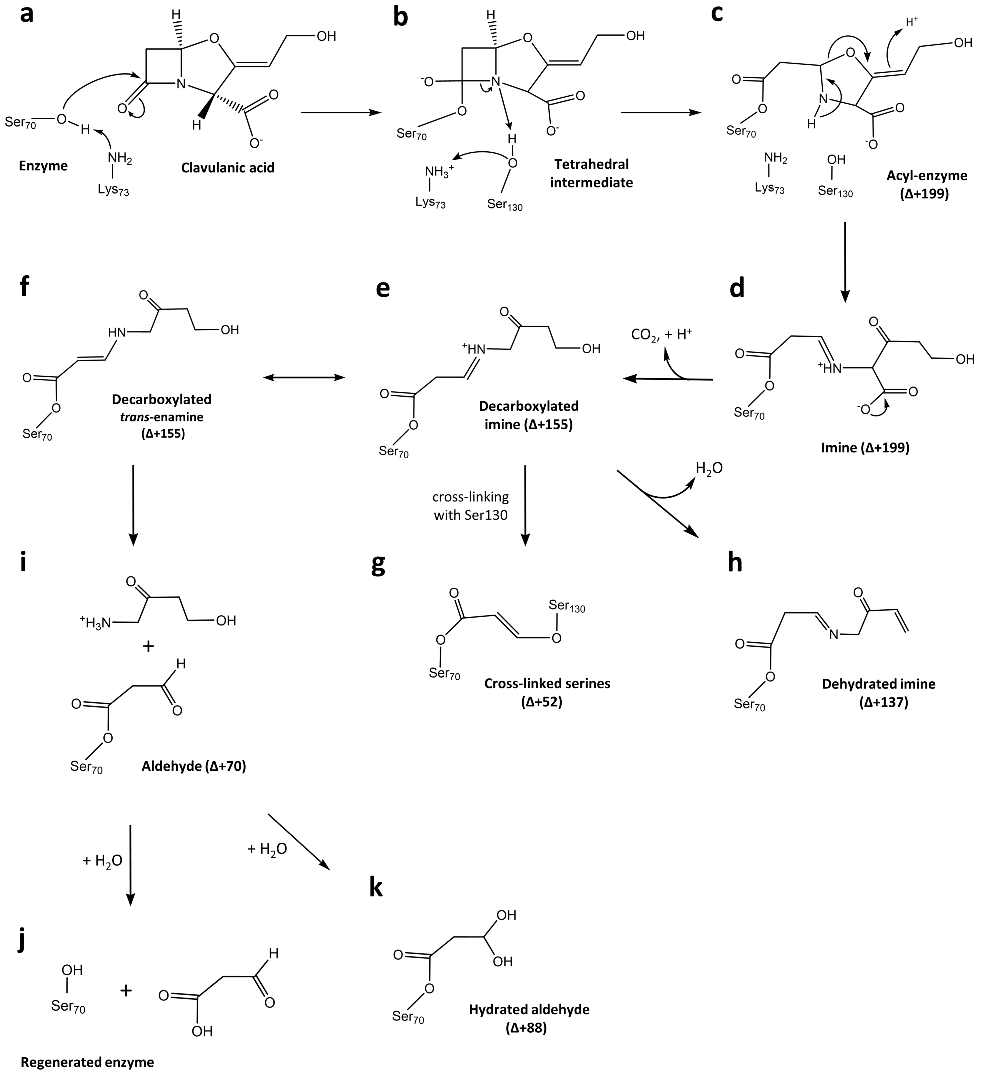

Figure S3. The proposed mechanism of BlaC inhibition by clavulanate and subsequent recovery. (a-k) The reaction scheme with all covalent adducts and the regenerated enzyme. Adapted from references. [2–4]

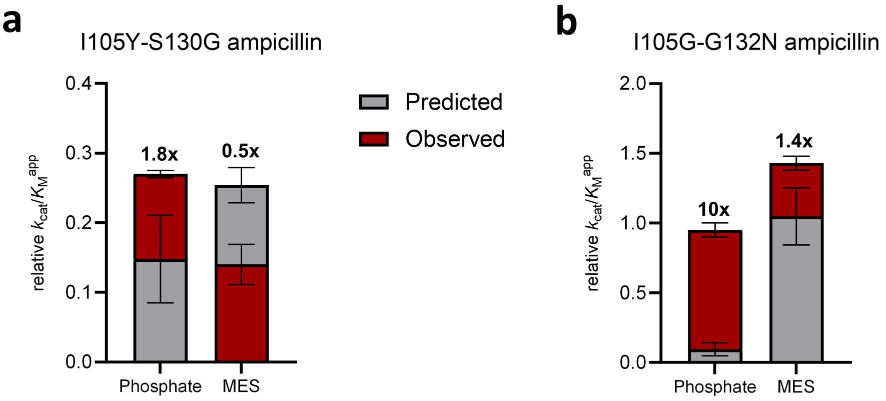

Figure S4. Difference in the magnitude of epistasis in enzyme activity between phosphate and MES buffer. Relative catalytic efficiency (k_{cat}/K_M^{app}) of the double mutants is plotted against predicted relative catalytic efficiencies from the k_{cat}/K_M^{app} values of the single mutants. If the difference is nonzero (including errors), epistasis occurs (see Methods in the main text for the detailed description). Numbers above bars represent fold differences between observed and predicted catalytic efficiencies, with values >1 indicating positive epistasis, and values <1 indicating negative epistasis. a – BlaC I105Y-S130G; b – BlaC I105G-G132N.

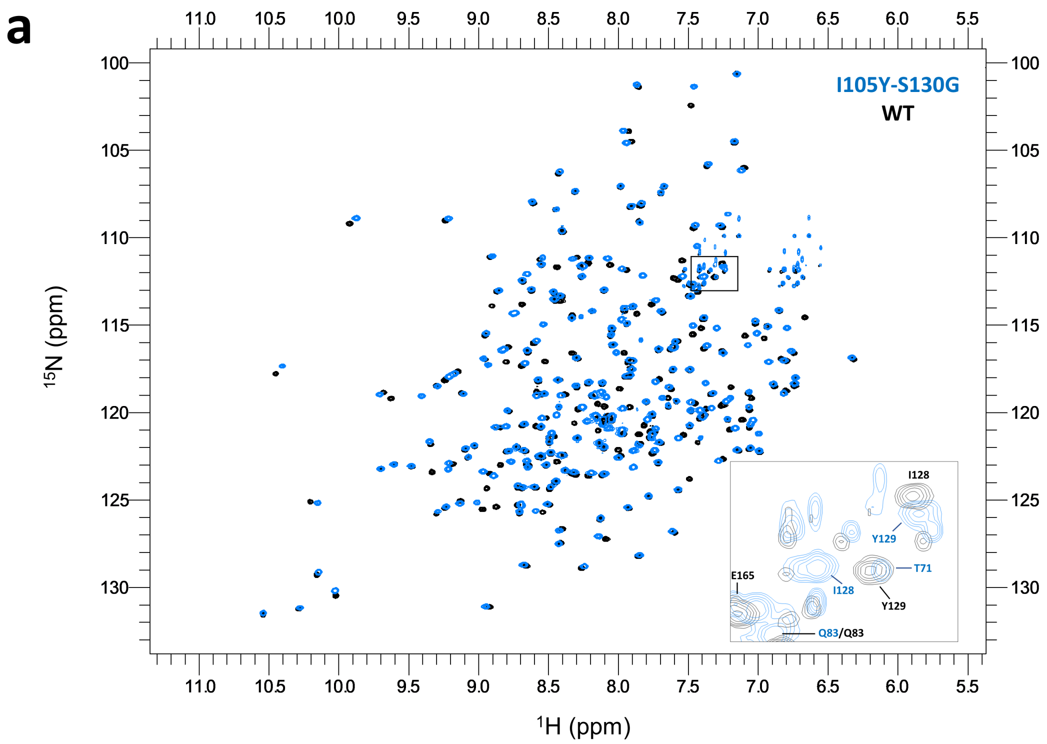

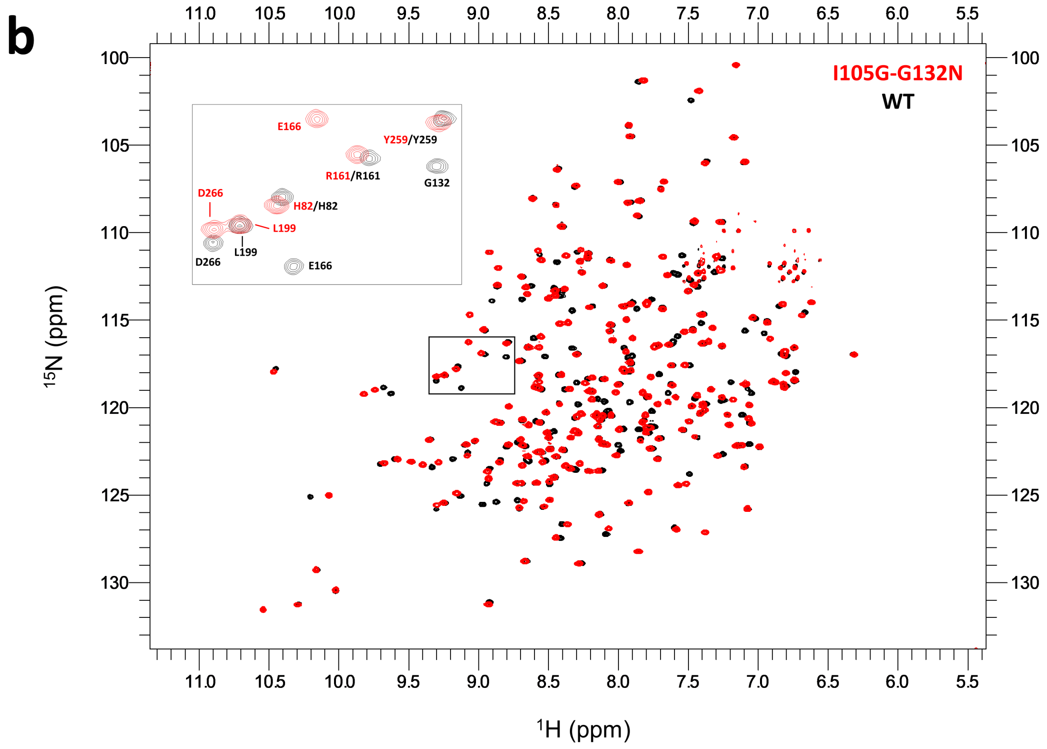

Figure S5. Overlay of ^{1}H-^{15}N TROSY-HSQC spectra for both double mutants and wild-type BlaC. (a) I105Y-S130G (blue), and wild-type (black). In the lower right corner, shown is the part of the spectrum with possible peak splitting of Tyr129 of the double mutant. (b) I105G-G132G (red), and wild-type (black). In the upper left corner, shown is the part of the spectrum where Glu166 shifts significantly compared to the wild-type enzyme.

```
1         10         20         30         40         50         60
MGSSHHHHHH SSGLVPRGSH MENLYFQSGG DLADRFAELE RRYDARLGVY VPATGTTAAI
                              ↑    30         40         50

        70         80         90        100        110        120
EYRADERFAF CSTFKAPLVA AVLHQNPLTH LDKLITYTSD DIRSISPVAQ QHVQTGMTIG
 60         70         80      90         100        110        120

       130        140        150        160        170        180
QLCDAAIRYS DGTAANLLLA DLGGPGGGTA AFTGYLRSLG DTVSRLDAEE PELNRDPPGD
         130        140            150        160        170

       190        200        210        220        230        240
ERDTTTPHAI ALVLQQLVLG NALPPDKRAL LTDWMARNTT GAKRIRAGFP ADWKVIDKTG
   180        190        200        210        220        230

       250        260        270        280        290
TGDYGRANDI AVVWSPTGVP YVVAVMSDRA GGGYDAEPRE ALLAEAATCV AGVLA
  240        250       260         270        280        290
```

Figure S6. Amino acid sequence of BlaC in pET28a used for the production of recombinant protein and *in vitro* characterization. The upper numbering corresponds to the actual sequence, the lower to the Ambler notation [5]. The N-terminal cleavable 6xHis tag is shown in green, and the TEV cleavage sequence in brown, with the arrow denoting the position where the cleavage occurs. The BlaC sequence starts at residue 28 (Ambler notation).

```
1         10         20         30         40         50         60
MANNDLFQAS RRRFLAQLGG LTVAGMLGPS LLTPRRATAA QADLADRFAE LERRYDARLG
                                                  30         40

        70         80         90        100        110        120
VYVPATGTTA AIEYRADERF AFCSTFKAPL VAAVLHQNPL THLDKLITYT SDDIRSISPV
    50         60         70         80        90        100

       130        140        150        160        170        180
AQQHVQTGMT IGQLCDAAIR YSDGTAANLL LADLGGPGGG TAAFTGYLRS LGDTVSRLDA
 110        120        130        140            150        160

       190        200        210        220        230        240
EEPELNRDPP GDERDTTTPH AIALVLQQLV LGNALPPDKR ALLTDWMARN TTGAKRIRAG
    170        180        190        200        210        220

       250        260        270        280        290        300
FPADWKVIDK TGTGDYGRAN DIAVVWSPTG VPYVVAVMSD RAGGGYDAEP REALLAEAAT
    230        240        250       260         270        280

       310
CVAGVLALEH HHHHH
   290
```

Figure S7. Amino acid sequence of BlaC in pUK21 used for minimum inhibitory concentration (MIC) determinations. The upper numbering corresponds to the actual sequence, the lower to the Ambler notation [5]. The N-terminal twin-arginine translocation signal peptide is indicated in red, and the C-terminal 6xHis tag with two additional residues (LE) is indicated in green.

SUPPLEMENTARY REFERENCES

1. Radojković, M. *et al.* A glycine at position 105 leads to clavulanic acid and avibactam resistance in class A β-lactamases. *J Biol Chem* **301**, 110347 (2025).

2. Drawz, S. M. & Bonomo, R. A. Three decades of β-lactamase inhibitors. *Clin Microbiol Rev* **23**, 160–201 (2010).

3. Hugonnet, J. E. & Blanchard, J. S. Irreversible inhibition of the Mycobacterium tuberculosis β-lactamase by clavulanate. *Biochemistry* **46**, 11998–12004 (2007).

4. Tassoni, R., Blok, A., Pannu, N. S. & Ubbink, M New conformations of acylation adducts of inhibitors of β-lactamase from Mycobacterium tuberculosis. *Biochemistry* **58**, 997-1009 (2019)

5. Ambler, R. P. *et al.* A standard numbering scheme for the class A β-lactamases. *Biochem J* **276**, 269–270 (1991).
